# Prevalence and Genomic Investigation of Multidrug-Resistant *Salmonella* Isolates from Companion Animals in Hangzhou, China

**DOI:** 10.3390/antibiotics11050625

**Published:** 2022-05-05

**Authors:** Lin Teng, Sihao Liao, Xin Zhou, Chenghao Jia, Mengyao Feng, Hang Pan, Zhengxin Ma, Min Yue

**Affiliations:** 1 Department of Veterinary Medicine, Institute of Preventive Veterinary Sciences, College of Animal Sciences, Zhejiang University, Hangzhou 310058, China; tenglinchn@zju.edu.cn (L.T.); 22017106@zju.edu.cn (S.L.); zxin@zju.edu.cn (X.Z.); 22017118@zju.edu.cn (C.J.); 22017123@zju.edu.cn (M.F.); 11817015@zju.edu.cn (H.P.); 2 Mount Desert Island Biological Laboratory, Bar Harbor, ME 04609, USA; zma@mdibl.org; 3 Zhejiang Provincial Key Laboratory of Preventive Veterinary Medicine, Hangzhou 310058, China

**Keywords:** *Salmonella*, pets, prevalence, antimicrobial resistance, whole genome sequencing

## Abstract

*Salmonella* is a group of bacteria that constitutes the leading cause of diarrheal diseases, posing a great disease burden worldwide. There are numerous pathways for zoonotic *Salmonella* transmission to humans; however, the role of companion animals in spreading these bacteria is largely underestimated in China. We aimed to investigate the prevalence of *Salmonella* in pet dogs and cats in Hangzhou, China, and characterize the antimicrobial resistance profile and genetic features of these pet-derived pathogens. In total, 137 fecal samples of pets were collected from an animal hospital in Hangzhou in 2018. The prevalence of *Salmonella* was 5.8% (8/137) in pets, with 9.3% (5/54) of cats and 3.6% (3/83) of dogs being *Salmonella* positive. By whole-genome sequencing (WGS), in silico serotyping, and multilocus sequence typing (MLST), 26 pet-derived *Salmonella* isolates were identified as *Salmonella* Dublin (ST10, *n* = 22) and *Salmonella* Typhimurium (ST19, *n* = 4). All of the isolates were identified as being multidrug-resistant (MDR), by conducting antimicrobial susceptibility testing under both aerobic and anaerobic conditions. The antibiotics of the most prevalent resistance were streptomycin (100%), cotrimoxazole (100%), tetracycline (96.20%), and ceftriaxone (92.30%). Versatile antimicrobial-resistant genes were identified, including *flo*R (phenicol-resistant gene), *bla*CTX-M-15, and *bla*CTX-M-55 (extended-spectrum beta-lactamase genes). A total of 11 incompatible (Inc) plasmids were identified, with IncA/C2, IncFII(S), and IncX1 being the most predominant among *Salmonella* Dublin, and IncFIB(S), IncFII(S), IncI1, and IncQ1 being the most prevailing among *Salmonella* Typhimurium. Our study applied WGS to characterize pet-derived *Salmonella* in China, showing the presence of MDR *Salmonella* in pet dogs and cats with a high diversity of ARGs and plasmids. These data indicate a necessity for the regular surveillance of pet-derived pathogens to mitigate zoonotic diseases.

## 1. Introduction

Antimicrobial-resistant pathogens are great threats to human health, causing global morbidity and mortality [1]. To mitigate the effects of antimicrobial resistance, numerous studies have clearly documented antibiotic resistance pathogens in food animals, slaughterhouses, retail foods, and humans [2,3,4,5,6,7,8,9,10,11], whereas the exact role of pet-derived, antimicrobial-resistant pathogens is largely underestimated [12,13]. It was reported that about 60% of U.S. households owned at least one pet, with estimated numbers of 70.0 to 78.2 million dogs and 74.1 to 86.4 million cats [14]. The number of pet dogs in the U.K. was about 9 million in 2014 [15]. In China, the pet population annually increased by 7.0%, with estimated pet dog and cat numbers of 136.1 million and 171.2 million in 2022, respectively [16]. As a large population of people begins to raise pets and consider them family members, the chances of direct contact between pets and humans increases, placing humans under the threat of antimicrobial-resistant and pet-derived pathogens, including zoonotic *Salmonella* [17,18,19].

*Salmonella* consists of more than 2500 serovars and causes more than 93.8 million illness cases and 155 thousand deaths annually [20,21]. These zoonotic foodborne bacteria have been identified in a wide range of hosts, including humans, cattle, swine, chickens, dogs, and cats [1,22]. Humans can be infected by *Salmonella* after ingesting farm products that have been contaminated by these bacteria, and consequently develop a series of clinical symptoms, including gastroenteritis, bacteremia, and enteric fever [23]. Although the zoonotic transmission of *Salmonella* between pets and humans has rarely been reported [24], the presence of MDR *Salmonella* was observed in pet dogs and cats from Thailand, the U.K., Italy, and Ethiopia [25,26,27], suggesting a high potential for interspecies transmission. However, the prevalence of *Salmonella* from pets in China has not been extensively investigated, indicating that it is essential to conduct surveillance for pet-derived *Salmonella*, to prevent the zoonotic dissemination of these pathogens.

Whole-genome sequencing (WGS) is a new gold standard for pathogen monitoring, providing insights into the source of pathogens and the genomic characteristics of bacteria [28]. Several studies have applied WGS to characterize the MDR *Escherichia* coli from pet dogs and cats [12,18,29], while only a limited number of studies have used WGS to investigate the genomic characteristics of the pet dog- and cat-derived *Salmonella* [24]. In the few studies available using WGS, *Salmonella* isolates from shelter dogs in Texas, U.S. were found to consist of a variety of *Salmonella* serovars (e.g., Newport and Javiana) and carry a range of antimicrobial-resistant genes (ARGs) [24]; a survey identified various *Salmonella* serovars (e.g., Typhimurium, Newport, and Javiana) from diarrheic and non-diarrheic dogs and cats in the U.S. [30]. There is currently a dearth of research on antibiotic resistance and the genomic characterization of pet-derived *Salmonella* in China.

In the current study, we investigated the prevalence and antibiotic resistance of pet-derived *Salmonella* in Hangzhou, one of the largest cities in China. WGS was performed to understand the genetic characteristics of these pathogens, including their serovars, sequence types, ARGs, and plasmid types.

## 2. Materials and Methods

### 2.1. Ethical Statement

A sample collection was conducted under the approval and supervision of the Zhejiang University Animal Ethics Committee and the approval document (ZJU20190094).

### 2.2. Sample Collection and Salmonella Isolation

The fecal samples of cats (*n* = 54) and dogs (*n* = 83) were collected in the Veterinary Hospital of Zhejiang University between March and December 2018. The isolation of *Salmonella* was performed following the method previously described [11,31]. Briefly, 10 g of feces were pre-enriched in 90 mL buffered peptone water (BPW) at 37 °C for 18–20 h. The enriched culture was then transferred to modified semisolid Rappaport Vassiliadis (MSRV) agar and incubated at 42 °C for 24 h. Then, the positive colonies on MRSV agar were inoculated on xylose lysine deoxycholate (XLD) agar plates and incubated for a further 18–20 h at 37 °C. Finally, typical black-centered colonies on XLD media were picked to confirm the *Salmonella* isolates, using Matrix-assisted laser desorption/ionization time-of-flight mass spectrometry (MALDI-TOF MS) [32,33]. The device and database were obtained from the MALDI Biotyper (Bruker Daltonics, Bremen, Germany). For each sample, up to five suspected *Salmonella* isolates were picked for MALDI-TOF MS. All of the *Salmonella* isolates that were confirmed using MALDI-TOF MS were picked for WGS.

### 2.3. Antimicrobial Susceptibility Testing

The antimicrobial resistance of the *Salmonella* isolates was phenotypically investigated using the broth microdilution method described previously [31]. In total, 15 antimicrobials belonging to 10 classes were used in this study, including kanamycin (KAN), gentamicin (GEN), streptomycin (STR), ampicillin (AMP), amoxicillin/clavulanic acid (AMC), ceftiofur (CF), ceftriaxone (CRO), cefoxitin (FOX), chloramphenicol (CHL), tetracycline (TET), azithromycin (AZM), cotrimoxazole (SXT), nalidixic acid (NAL), ciprofloxacin (CIP), and colistin (CST) (Table S2). The results were categorized according to the cut-off values recommended according to the CLSI guidelines (Table S2) [34]. The minimal inhibitory concentration (MIC) assay was conducted under both aerobic and anaerobic conditions. The MDR isolates were identified based on their resistance to 3 or more antimicrobial classes. *Pseudomonas aeruginosa* ATCC 27853 and *Escherichia coli* ATCC 25922 strains were used as quality controls to verify the validity of the results. Antimicrobial susceptibility testing was triplicated.

### 2.4. Genomic DNA Extraction and Whole-Genome Sequencing

The genomic DNA extraction and WGS were conducted as previously described [35]. Bacterial genomic DNA was extracted using a commercial TIANamp bacteria DNA kit (Tiangen Biotech, Beijing, China). Genomic DNA was used for DNA library construction, followed by WGS using the Illumina HiSeq platform, to generate paired-end reads of 150 bp.

### 2.5. Genome Assembly and Bioinformatic Analyses

Genome assembly and bioinformatic analyses were conducted as previously described, with minor modifications [36,37,38]. Low-quality sequences generated using the Illumina HiSeq were removed using Trimmomatic [39]. To acquire the whole genome sequences, raw reads were quality-checked and assembled using SPAdes v3.12.0 [40]. The in silico serotyping of *Salmonella* was performed using SISTR v1.0.2 [41]. The sequence type (ST) of the isolates was determined via MLST v2.16.1. Furthermore, antimicrobial resistance genes (ARGs) and plasmid types were detected using the Abricate v0.8 [42]. The ARGs were identified using the ResFinder, with a cut-off of 90% identity, and the plasmid type of each isolate was identified using the PlasmidFinder, with a cut-off of 95% identity [43].

### 2.6. Data Availability

Whole-genome sequences of 26 *Salmonella* isolates were submitted to the NCBI database and deposited under the BioProject PRJNA828007 (Table S1).

## 3. Results

### 3.1. Prevalence of Salmonella Isolates in Companion Animals

A total of 26 *Salmonella* isolates were obtained from 5.8% (8/137) of fecal samples of companion animals, with 9.3% (5/54) in cats and 3.6% (3/83) in dogs (Table 1). The prevalence of *Salmonella* in pets under one year old and over one year old were 8.2% (5/61) and 3.9% (3/76), respectively (Table 1). A higher prevalence of *Salmonella* was observed in pets with intestinal diseases than in pets with other diseases or healthy pets, i.e., 9.4% in pets with intestinal diseases, 1.9% in pets with other diseases, and 5.3% in healthy pets (Table 1). Interestingly, no *Salmonella* was recovered from the pets that had undergone antibiotic treatment in the previous month, while 7.1% (8/112) of pets without antibiotic treatment carried *Salmonella*.

### 3.2. Phenotypic Antimicrobial Resistance

**Figure 1:**
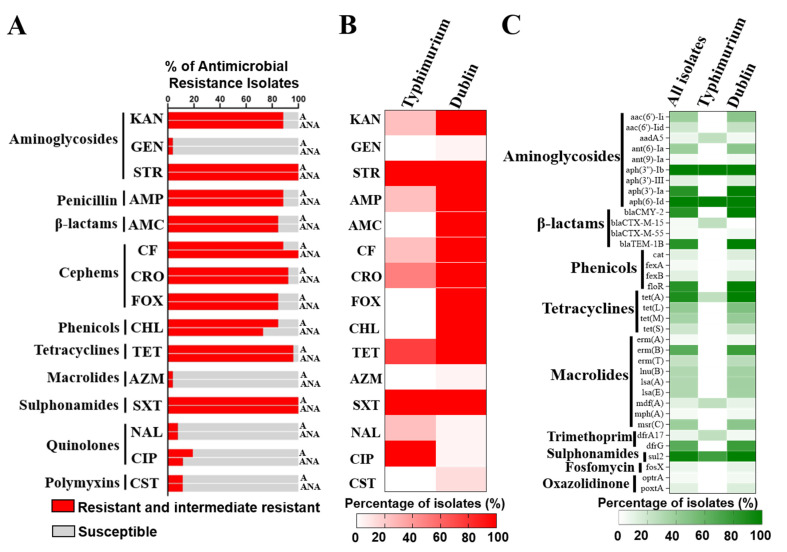
The antimicrobial resistance of companion-animal-origin *Salmonella* isolates. (**A**) Antimicrobial-resistant phenotypes under aerobic and anaerobic conditions. A total of 15 antibiotics were used, including kanamycin (KAN), gentamicin (GEN), streptomycin (STR), ampicillin (AMP), amoxicillin/clavulanic acid (AMC), ceftiofur (CF), ceftriaxone (CRO), cefoxitin (FOX), chloramphenicol (CHL), tetracycline (TET), azithromycin (AZM), cotrimoxazole (SXT), nalidixic acid (NAL), ciprofloxacin (CIP), and colistin (CST). The results of MIC under aerobic (“A”) and anaerobic (“ANA”) conditions are displayed. (**B**) Heatmap of antimicrobial-resistant phenotypes of distinct *Salmonella* serovars. (**C**) Heatmap of antimicrobial-resistant genes carried by *Salmonella*.

Pet-derived MDR *Salmonella* poses threats to the health of humans; therefore, we investigated the phenotypic antimicrobial resistance of these pet-derived *Salmonella* isolates under aerobic conditions, using 15 antibiotics from 10 antibiotic classes (Figure 1A). A high percentage of isolates were resistant to STR (100%), SXT (100%), TET (96.20%), CRO (92.30%), KAN (88.50%), AMP (88.50%), CF (88.50%), AMC (84.60%), FOX (84.60%), and CHL (84.60%), while a low proportion of isolates showed resistance to GEN (3.80%), AZM (3.80%), NAL (7.70%), CIP (11.54%), and CST (11.50%). Strikingly, all *Salmonella* isolates were resistant to 3 to 10 classes of antibiotics, indicating that all strains were MDR (Table S3). Since the intestinal tract is an anaerobic environment and these pathogens colonize in such an environment, we further conducted MIC in the anaerobic condition to understand whether the anaerobic condition affects the antimicrobial susceptibility of these isolates (Figure 1A). The results showed that 96.2% (25/26) of the strains were MDR (Table S4). In both the aerobic and anaerobic conditions, the same proportions of isolates were resistant to all tested antibiotics except CF, CHL, and CIP. Compared with the aerobic condition, more isolates were resistant to CF (100.0% vs. 88.5%), and less isolates were resistant to CHL (73.1% vs. 84.6%) and CIP (11.54% vs. 19.2%) under anaerobic conditions (Figure 1A).

By whole-genome sequencing, these *Salmonella* isolates were identified as *Salmonella* Dublin (84.6%; 22/26) and *Salmonella* Typhimurium (15.4%; 4/26). The *Salmonella* Dublin isolates displayed a broader antimicrobial-resistant spectrum than the *Salmonella* Typhimurium isolates. Resistance to GEN, AMC, FOX, CHL, AZM, and CST was only found in the *Salmonella* Dublin isolates, while the resistance to NAL and CIP was only observed in the *Salmonella* Typhimurium isolates (Figure 1B).

### 3.3. Antimicrobial Resistance Genes

To investigate the antimicrobial resistance gene profiles of these *Salmonella* isolates, we detected the ARGs of these *Salmonella* Dublin (ST10) and *Salmonella* Typhimurium (ST19) isolates (Table S1). In total, 41 different ARGs belonging to eight antibiotic classes were identified in the 26 *Salmonella* isolates (Figure 1C). All of the *Salmonella* isolates carried *aph*(3″)-*Ib* and *aph*(6)-*Id* (aminoglycosides resistant genes). The majority of *Salmonella* Typhimurium also carried *sul*2 (sulphonamide resistance gene), besides *aph*(3″)-*Ib* and *aph*(6)-*Id.* Compared with *S.* Typhimurium, the *Salmonella* Dublin isolates carried 27 more ARGs. Most of the *S.* Dublin isolates carried *aph*(3′)-*Ia*, *aph*(3″)-*Ib*, *aph*(6)-*Id* (aminoglycoside resistance genes), *bla*CMY-2, *bla*TEM-1B (β-lactamase genes), *flo*R (phenicol-resistant gene), *tet*(*A*) (tetracycline-resistant gene), and *sul*2.

### 3.4. Versatile Plasmids in Pet-Derived Salmonella

**Figure 2:**
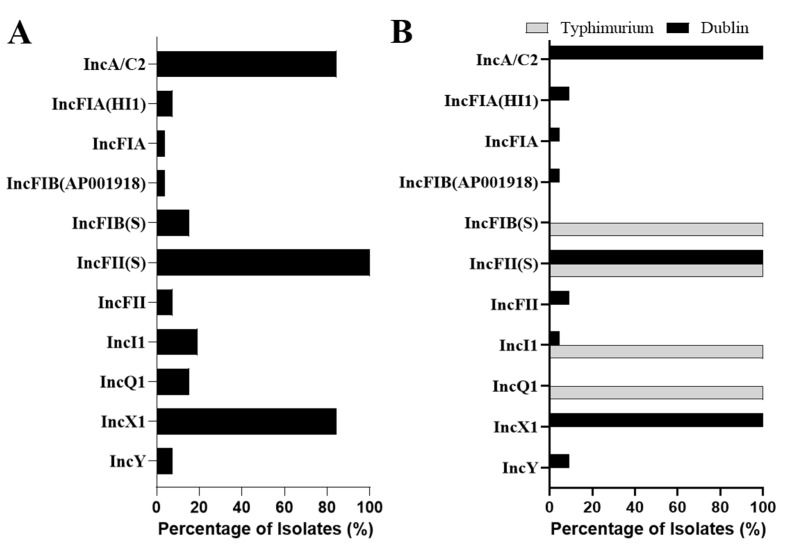
Versatile plasmid replicon types identified in companion-animal-origin *Salmonella* isolates. (**A**) Percentage of *Salmonella* isolates carrying diverse plasmid replicon types. (**B**) Distribution of plasmid replicon types in *Salmonella* Dublin and *Salmonella* Typhimurium.

Plasmids can disseminate ARGs through horizontal gene transfer. To identify the plasmid carried by these pet-derived *Salmonella* isolates, we detected the plasmid types of each isolate iin silico. A total of 11 plasmids of incompatibility (Inc) groups were detected, with the IncFII(S) (100%; 26/26), IncA/C2 (85%; 22/26), and IncX1 (85%; 22/26) plasmids being the most prevalent (Figure 2A). *Salmonella* Dublin showed a higher diversity of Inc plasmids than *S.* Typhimurium. IncA/C2 and IncX1 were only maintained by *Salmonella* Dublin, while the IncFIB(S) and IncQ1 plasmids were conserved and unique in *Salmonella* Typhimurium (Figure 2B).

## 4. Discussion

In this study, we investigated the prevalence of *Salmonella* isolates in pet dogs and cats in Hangzhou, China, finding that 9.3% of cats and 3.6% of dogs carried these bacteria (Table 1). Yang et al. reported that the prevalence of *Salmonella* was 7.08% in pet dogs, and 2.31% in pet cats in Xuzhou, China [44]. These numbers are relatively low compared with the prevalence of *Salmonella* in dogs in other countries [26,45,46]. A study in Ethiopia showed that samples from 11.0% of dogs contained *Salmonella*, after examining 360 dogs [26]. The prevalence of *Salmonella* in dogs was 12.9% (18/140) from a study in Thailand [45]. Another study from Canada reported that 23.2% (32/138) of dogs had *Salmonella* in their feces [46]. The prevalence of *Salmonella* in pet dogs varied across studies and counties, indicating that the prevalence is affected by multiple risk factors. In the current study, pets with intestinal diseases showed a higher rate (9.4%) of *Salmonella* carriage than those with other diseases (1.9%) and with a healthy condition (5.3%) (Table 1), suggesting that the presence of *Salmonella* in the animals may be associated with their health condition. Pets younger than 1 year of age showed a higher prevalence of *Salmonella* (Table 1), which may be explained by their immature gut microbiota or immune system [47,48,49]. Other risk factors contributing to the carriage of *Salmonella* include events such as consuming raw food, contacting livestock, receiving a probiotic, and eating a raw food diet [46].

The zoonotic transmission of pathogens from pets to their owners poses a threat to human health. The pet-derived *Salmonella* isolates identified in this study are *Salmonella* Dublin (ST10) and *Salmonella* Typhimurium (ST19) (Table S1). *Salmonella* Typhimurium isolates were detected in pet dogs or cats in several studies in Thailand, South Africa, China, and the U.K. [1,22,25,44]. It was one of the predominant serovars from stray dogs at the U.S.–Mexico border [50]. Compared with *Salmonella* Typhimurium, *Salmonella* Dublin isolates were less frequently identified in companion animals. The only study available discovered that both serovars from dogs and cats were documented in the U.K. [25]. Although no direct evidence confirms the transmission of *Salmonella* from pets to humans, pets and humans in the same household were reported to share clonal pathogenic *E. coli* isolates [17,18], suggesting plausible zoonotic transmission.

The pet diet is one of the major sources of pet-derived bacteria. Feeding pets with raw meat-based diets (RMBDs) has become a trend in many developed countries [51]. A recent study tested 35 RMBD products from eight brands, finding a range of zoonotic bacteria, including *Salmonella* species, *Escherichia coli* serotype O157:H7, and *Listeria monocytogenes* [52]. Similarly, Strohmeyer et al. revealed the contamination of commercially available diets for dogs by *Salmonella enterica* (5.9%, 17/288), and non-type-specific *Escherichia coli* (53.0%, 153/288), after investigating 288 samples of raw meat diets, dry dog foods, and canned dog foods [53]. Additionally, *Salmonella*-contaminated pet treats, including beef, pig ear, and seafood, were reported to cause outbreaks of human illness or human infection in Canada and the U.S. [54,55,56]. *Salmonella* Dublin is host-adapted to cattle [57,58]. Our previous study found that the carriage of the IncA/C2 plasmid is a typical feature of the bovine-derived *Salmonella* Dublin (ST10) isolates [59]. Interestingly, all of the pet-derived *Salmonella* Dublin (ST10) isolates in this study carried IncA/C2 (Figure 2B). Although we did not investigate bacteria in pet diets, the presence of *Salmonella* Dublin in pet feces might be associated with the ingestion of beef-based diets contaminated by this bacterium. Collectively, these data suggest that bacteria contamination is common in commercially available RMBDs, which pose risks to the health of humans and pets.

All of the pet-derived *Salmonella* isolates were MDR and carried a variety of ARGs (Figure 1 and Table S3). Notably, in the genomic sequences of pet-derived *Salmonella* isolates, we identified *bla*CTX-M-15, *bla*CTX-M-55, and *bla*CMY-2 genes conferring bacterial resistance to the third-generation cephalosporins that are widely used in human clinics to treat the infection of Gram-negative bacteria. Consistent with our study, *bla*CTX-M-55 was identified in *Salmonella* Stockholm from pet dogs and cats in Thailand [60], indicating that third-generation cephalosporin-resistant bacteria are widely spread in companion animals. Importantly, the carriage of *flo*R, which confers phenicol resistance, was seldom reported in pet-derived *Salmonella*, but all of our *Salmonella* Dublin isolates carried this gene, showing a potential lack of information (Figure 2C). The presence of these ARGs (e.g., *bla*CTX-M-15, *bla*CTX-M-55, *bla*CMY-2, and *flo*R) in *Salmonella* from pet dogs and cats was rarely recorded, which may be explained by the lack of WGS-based surveillance for the pet-derived bacteria.

In summary, the MDR *Salmonella* Typhimurium and Dublin were identified from pet dogs and cats in Hangzhou, China. This straightforward genomic characterization demonstrated that pet-derived *Salmonella* isolates carry a range of ARGs and plasmids. Our findings highlight the potential role of pet dogs and cats as carriers of MDR *Salmonella*, posing a risk of zoonotic transmission with enormous public health concerns. Further investigation into pet diets can help to reveal the source of the pet-derived *Salmonella*. Therefore, it is necessary to conduct regular surveillance for bacteria in pets and pet diets, and to educate pet owners about proper hygiene practices in pet care.

## Figures and Tables

**Table 1:** Prevalence of *Salmonella* in distinct pet categories.

Category	Sub-Category (Number of Animals)	Number of Animals Containing *Salmonella*	Prevalence of *Salmonella*
Host	All Pets (*n* = 137)	8	5.8%
	Cat (*n* = 54)	5	9.3%
	Dog (*n* = 83)	3	3.6%
Health condition	Healthy pets (*n* = 19)	1	5.3%
	Pets with intestinal disease (*n* = 64)	6	9.4%
	Pets with other disease (*n* = 54)	1	1.9%
Animal Age	<1 year old (*n* = 61)	5	8.2%
	≥1 year old (*n* = 76)	3	3.9%
Antibiotic treatment in the previous month	Yes (*n* = 25)	0	0.0%
	No (*n* = 112)	8	7.1%

## Data Availability

The data presented in this study are openly available in NCBI database under BioProject PRJNA828007.

## Supplementary Materials

The following supporting information can be downloaded at: https://www.mdpi.com/article/10.3390/antibiotics11050625/s1, Table S1: A summary of the companion-animal-origin *Salmonella* isolates; Table S2: Antimicrobial susceptibility testing of *Salmonella* isolates; Table S3: The antimicrobial-resistant pattern of *Salmonella* isolates in the aerobic condition; Table S4. The antimicrobial-resistant pattern of *Salmonella* isolates in the anaerobic condition.
