# Supplementary material for: Prevalence and Genomic Investigation of Multidrug-Resistant Salmonella Isolates from Companion Animals in Hangzhou, China
Source: Antibiotics (Basel). 2022 May 5;11(5):625. doi: 10.3390/antibiotics11050625 (PMC9137667; doi:10.3390/antibiotics11050625)
Supplement: Supplementary file 1 [file antibiotics-11-00625-s001.zip › antibiotics-1715495-supplementary.pdf]

Supplementary Table S1. A summary of the companion-animal-origin *Salmonella* isolates.

Strain name	Serotype	Sequencing type	BioProject number	BioSample number
20180713-3-4	Typhimurium	ST19	PRJNA828007	SAMN27655659
20180713-3-6	Typhimurium	ST19	PRJNA828007	SAMN27655660
20180713-3-7	Typhimurium	ST19	PRJNA828007	SAMN27655661
20180713-3-11	Typhimurium	ST19	PRJNA828007	SAMN27655662
20181014-1-2	Dublin	ST10	PRJNA828007	SAMN27655663
20181017-1-6	Dublin	ST10	PRJNA828007	SAMN27655664
20181017-1-7	Dublin	ST10	PRJNA828007	SAMN27655665
20181017-2-1	Dublin	ST10	PRJNA828007	SAMN27655666
20181017-2-2	Dublin	ST10	PRJNA828007	SAMN27655667
20181017-2-5	Dublin	ST10	PRJNA828007	SAMN27655668
20181018-2-3	Dublin	ST10	PRJNA828007	SAMN27655669
20181018-2-4	Dublin	ST10	PRJNA828007	SAMN27655670
20181018-2-5	Dublin	ST10	PRJNA828007	SAMN27655671
20181018-2-7	Dublin	ST10	PRJNA828007	SAMN27655672
20181018-2-9	Dublin	ST10	PRJNA828007	SAMN27655673
20181018-3-2	Dublin	ST10	PRJNA828007	SAMN27655674
20181018-3-4	Dublin	ST10	PRJNA828007	SAMN27655675
20181018-3-7	Dublin	ST10	PRJNA828007	SAMN27655676
20181018-3-11	Dublin	ST10	PRJNA828007	SAMN27655677
20181018-3-13	Dublin	ST10	PRJNA828007	SAMN27655678
20181019-1-13	Dublin	ST10	PRJNA828007	SAMN27655679
20181019-1-17	Dublin	ST10	PRJNA828007	SAMN27655680
20181019-1-18	Dublin	ST10	PRJNA828007	SAMN27655681
20181019-2-3	Dublin	ST10	PRJNA828007	SAMN27655682
20181019-2-4	Dublin	ST10	PRJNA828007	SAMN27655683
20181019-2-9	Dublin	ST10	PRJNA828007	SAMN27655684

Supplementary Table S2. Antimicrobial susceptibility testing of *Salmonella* isolates.

Class	Antibiotic agent	Abbreviation	Antibiotic concentration range (μg/ml)	Breakpoint interpretive criteria (μg/mL)			%AR (aerobic)	%AR (anaerobic)
				S*	I	R		
Aminoglycosides	Kanamycin	KAN	0.125~64	≤16	32	≥64	88.50 %	88.50%
	Gentamicin	GEN	0.06~32	≤4	8	≥16	3.80%	3.80%
	Streptomycin	STR	0.125~64	≤16	-	≥32	100.00 %	100.00%
Penicillin	Ampicillin	AMP	0.25~128	≤8	16	≥32	88.50 %	88.50%
β-lactams combination	Amoxicillin-Clavulanic acid	AMC	64/32~0.12/0.06	≤8/4	16-32	≥32/16	84.60 %	84.60%
Cephems	Ceftiofur	CF	0.25~128	≤1	2	≥4	88.50 %	100.00%
	Ceftriaxone	CRO	0.25~128	≤1	2	≥4	92.30 %	92.30%
	Cefoxitin	FOX	0.125~64	≤8	16	≥32	84.60 %	84.60%
Phenicols	Chloramphenicol	CHL	0.125~64	≤8	16	≥32	84.60 %	73.10%
Tetracyclines	Tetracycline	TET	0.125~64	≤4	8	≥16	96.20 %	96.20%
Macrolides	Azithromycin	AZM	0.125~64	≤16	-	≥32	3.80%	3.80%
Sulphonamides	Cotrimoxazole	SXT	0.06/1.14~32/608	≤2/38	-	≥4/76	100.00 %	100.00%
Quinolones	Nalidixic acid	NAL	0.125~64	≤16	-	≥32	7.70%	7.70%
	Ciprofloxacin	CIP	0.03~16	≤1	2	≥4	19.20 %	11.54%
Polymyxins	Colistin	CST	0.06~32	≤2	-	≥4	11.50 %	11.50%

* S, sensitive; I, intermediate resistance; and R, resistant.

Supplementary Table S3. The antimicrobial-resistant pattern of *Salmonella* isolates in the aerobic condition.

No. of drugs	No. of antimicrobial classes	Antimicrobial-resistant spectrum*	No. of isolates
3	3	STR-SXT-CIP	1
4	4	STR-TET-SXT-CIP	1
5	5	STR-CRO-TET-SXT-CIP	1
9	6	KAN-STR-AMP-CF-CRO-TET-SXT-NAL-CIP	1
10	7	KAN-STR-AMP-AMC-CF-CRO-FOX-CHL-TET-SXT	19
11	8	KAN-STR-AMP-AMC-CF-CRO-FOX-CHL-TET-SXT-CST	1
12	9	KAN-STR-AMP-AMC-CF-CRO-FOX-CHL-TET-SXT-CIP-CST	1
14	10	KAN-GEN-STR-AMP-AMC-CF-CRO-FOX-CHL-TET-AZM-SXT-NAL-CST	1

* KAN: Kanamycin, GEN: Gentamicin, STR: Streptomycin, AMP: Ampicillin, AMC: Amoxicillin/clavulanic acid, CF: Ceftiofur, CRO: Ceftriaxone, FOX: Cefoxitin, CHL: Chloramphenicol, TET: Tetracycline, AZM: Azithromycin, SXT: Cotrimoxazole, NAL: Nalidixic acid, CIP: Ciprofloxacin, CST: Colistin.

Supplementary Table S4. The antimicrobial-resistant pattern of *Salmonella* isolates in the anaerobic condition.

No. of drugs	No. of antimicrobial classes	Antimicrobial-resistant spectrum in anaerobic condition *	No. of isolates
2	2	STR-SXT	1
3	3	STR-TET-SXT	1
4	4	STR-CRO-TET-SXT	1
9	6	KAN-STR-AMP-AMC-CF-CRO-FOX-TET-SXT	3
9	6	KAN-STR-AMP-CF-CRO-TET-SXT-NAL-CIP	1
10	7	KAN-STR-AMP-AMC-CF-CRO-FOX-CHL-TET-SXT	16
11	8	KAN-STR-AMP-AMC-CF-CRO-FOX-CHL-TET-SXT-CST	1
12	9	KAN-STR-AMP-AMC-CF-CRO-FOX-CHL-TET-SXT-CIP-CST	1
14	10	KAN-GEN-STR-AMP-AMC-CF-CRO-FOX-CHL-TET-AZM-SXT-NAL-CST	1

* KAN: Kanamycin, GEN: Gentamicin, STR: Streptomycin, AMP: Ampicillin, AMC: Amoxicillin/clavulanic acid, CF: Ceftiofur, CRO: Ceftriaxone, FOX: Cefoxitin, CHL: Chloramphenicol, TET: Tetracycline, AZM: Azithromycin, SXT: Cotrimoxazole, NAL: Nalidixic acid, CIP: Ciprofloxacin, CST: Colistin .
